# Pilot study of epigenetic aging and treatment response to semaglutide in the SLIM LIVER study

**DOI:** 10.1038/s41514-026-00383-9

**Published:** 2026-04-21

**Authors:** Michael J. Corley, Alina P. S. Pang, Douglas W. Kitch, Amy Kantor, Fred Sattler, Pablo F. Belaunzaran-Zamudio, Todd T. Brown, Alan Landay, Jordan E. Lake, Kristine M. Erlandson

**Affiliations:** 1 University of California San Diego, Department of Medicine, Division of Geriatrics, San Diego, CA USA; 2 Harvard T.H. Chan School of Public Health, Boston, MA USA; 3 University of Southern California Keck School of Medicine, Los Angeles, CA USA; 4 National Institute of Allergy and Infectious Diseases (contractor), Rockville, MD USA; 5 John Hopkins University School of Medicine, Baltimore, MD USA; 6 University of Texas Medical Branch, Galveston, TX USA; 7 UTHealth Houston, Department of Medicine, Division of Infectious Diseases, Houston, TX USA; 8 University of Colorado Anschutz Medical Campus, Aurora, CO USA

**Keywords:** Biomarkers, Diseases, Medical research

## Abstract

Semaglutide, a GLP-1 receptor agonist, improves metabolic health and reduces liver fat in people with HIV (PWH) and metabolic dysfunction-associated steatotic liver disease (MASLD). This post hoc analysis of the 24-week SLIM LIVER single-arm trial (ACTG A5371, No. NCT04216589, registered 02nd Jan 2020) in 41 PWH with MASLD receiving semaglutide (1.0 mg weekly) aimed to evaluate its effect on epigenetic aging and determine whether changes in epigenetic clocks associate with clinical responsiveness. Over 24 weeks, we observed DunedinPACE median change +0.018 (IQR –0.023 to +0.053), PCDNAmTL –0.006 kb (IQR –0.073 to +0.054), and PCGrimAge +0.54 years (IQR –0.33 to +1.26). Participants with decreased DunedinPACE (41.5%) showed greater liver fat reduction (*p* = 0.024) and trend towards improved gait speed (*p* = 0.081). Increased PCDNAmTL was associated with better gait speed (*p* = 0.012). These data suggest early signals of semaglutide responsiveness and relationships to epigenetic age biomarkers. Epigenetic biomarkers may enhance precision in GLP-1RA therapy and enable noninvasive monitoring of biological aging. Trial Registration: ClinicalTrials.gov ID: NCT04216589, registered 02nd Jan 2020.

## Introduction

Glucagon-like peptide-1 receptor agonists (GLP-1 RAs), such as semaglutide, have emerged as a transformative class of therapeutics that promote weight loss, improve glycemic control, and provide systemic benefits across multiple organ systems^1–4^. Beyond their established role in diabetes management, GLP-1 RAs have demonstrated cardiovascular protection, potential neuroprotective effects, and improvement in inflammation and endothelial function, positioning them as promising therapeutic agents for the geroscience field^1–3,5–7^. The properties of GLP-1 RAs are particularly relevant for metabolic dysfunction–associated steatotic liver disease (MASLD), a leading comorbidity in people with HIV (PWH) characterized by excess intrahepatic triglyceride (IHTG) accumulation, insulin resistance, oxidative stress, and systemic inflammation^8^. While lifestyle-induced weight loss remains the cornerstone of MASLD management^9^, semaglutide^10^ has shown efficacy in reducing hepatic steatosis and improving broader metabolic parameters and physical function^11,12^. Such benefits may be especially important for PWH, who frequently experience an accelerated aging phenotype driven by chronic immune activation and metabolic dysregulation^13^.

Recent advances in epigenetic biomarker research have enabled the quantification of biological aging through DNA methylation-based “epigenetic clocks”^14^. These clocks serve as minimally invasive proxies of biological aging and have been linked to metabolic traits such as body mass index, visceral adiposity, insulin resistance, and liver disease severity^15–17^. In PWH, epigenetic age acceleration (EAA) is frequently observed, with estimates ranging from 3 to 7 years above chronological age^18–20^. Recent evidence suggests that EAA may also reflect liver disease progression. In a study of 325 individuals with MASLD, advanced fibrosis was associated with a 5% faster pace of aging, as measured by DunedinPACE, and a 10% reduction in telomere length, captured by DNAmTL, compared to those without fibrosis^17^. Similarly, in a separate study of individuals with biopsy-confirmed non-alcoholic steatohepatitis (NASH), EAA measured by the Horvath clock correlated with hepatic collagen content, though not fibrosis stage, and revealed differentially methylated CpG sites enriched in developmental and transcriptional regulatory pathways^16^. These findings underscore the potential of epigenetic aging measures as biomarkers of liver disease severity and therapeutic responsiveness. However, it remains unclear whether longitudinal changes in epigenetic biomarkers track with clinical improvements in response to interventions such as GLP-1 RA therapy.

To address this, we conducted a post hoc epigenetic analysis of participants from the SLIM LIVER study (Advancing Clinical Therapeutics Globally for HIV/AIDS and Other Infections (ACTG) A5371; NCT04216589), an open-label, single arm, Phase 2 clinical trial of semaglutide in PWH with MASLD. In the parent study, 24 weeks of low-dose semaglutide (1 mg subcutaneously weekly) significantly reduced IHTG by 31.3%, improved insulin sensitivity (approximately 1.5-unit decrease in Homeostatic Model Assessment of Insulin Resistance (HOMA-IR)), and lowered triglyceride levels by 27 mg/dL^12^. A secondary analysis also revealed preserved or improved physical function, with a significant reduction in the prevalence of slow gait speed ( < 1 m/s) despite modest muscle loss^11^. We hypothesized that within-individual changes in epigenetic aging biomarkers DunedinPACE, PCGrimAge, and DNAm telomere length (PCDNAmTL) over 24 weeks of semaglutide treatment would be associated with improvements in hepatic fat, metabolic markers, and physical function. This exploratory analysis aimed to assess the extent to which biological aging is modifiable in response to semaglutide and whether such changes are associated with therapeutic benefit in PWH with MASLD.

## Results

### SLIM LIVER epigenetic substudy characteristics

Characteristics of the overall SLIM LIVER cohort have been previously described^12^. Forty-one of 51 enrolled participants had evaluable samples at both time points for our post hoc exploratory epigenetic analysis (Table 1). The median age was 52 years (interquartile range [IQR]: 41–57.5). Obesity and central adiposity were common (by design), with a median BMI of 35 kg/m² (IQR: 31–39) and median waist circumference of 114 cm (IQR: 107–124). All participants were on suppressive antiretroviral therapy (ART) with HIV-1 RNA levels below 50 copies/mL, and the median CD4 + T-cell count was 701 cells/mm³ (IQR: 586–869). Metabolic parameters reflected elevated cardiometabolic risk, with a median HOMA-IR of 3.8 (IQR: 2.8–6.1) and fasting glucose of 98 mg/dL (IQR: 93–107). Median fasting triglycerides were 116 mg/dL (IQR: 95–183), and alanine aminotransferase (ALT) was elevated in 53% of participants. ART regimens were predominantly integrase strand transfer inhibitor (INSTI)-based (82%), with smaller proportions on non-nucleoside reverse transcriptase inhibitor (NNRTI; 22%)- or protease inhibitor (PI; 4%)-based regimens.

**Table 1:** Baseline participant characteristics

Baseline characteristic	Groups	All participants (*N* = 41)	IHTG% Change Mean (SD)
Overall			−28.8 (27.3)
Age (years)		52 (41.0, 57.5)	
	<50	18 (44%)	−24.4 (28.5)
	>50	23 (56%)	−32.2 (26.5)
Natal sex	Male	25 (61%)	−24.1 (30.0)
	Female	16 (39%)	−36.2 (21.3)
Race/Ethnicity	White non-Hispanic	13 (27%)	
	Black	16 (33%)	
	Hispanic	19 (39%)	
	American Indian/ Alaska Native	1 (2%)	
BMI (kg/m^2^)		35 (31, 39)	
HIV-1 RNA (copies/mL)	<20	30 (73%)	−27.3 (27.8)
	<50	11 (27%)	−33.0 (26.9)
CD4 count (cells/mm³)		701 (606, 848)	
	<500	5 (12%)	−31.5 (34.5)
	≥500	36 (78%)	−28.4 (26.8)
CD4/CD8 ratio (cells/mm³)		1.21 (0.714, 1.72)	
	<1	12 (29%)	−33.1 (26.0)
	≥1	29 (71%)	−27.0 (28.1)
ART Regimen			
PI		2 (4%)	
NNRTI		10 (22%)	
INSTI		40 (82%)	

*IHTG* intrahepatic triglyceride, *BMI* body mass index, *ART* antiretroviral therapy; regimen classes indicate the presence of an agent from that class at baseline and are not mutually exclusive (NRTI backbones not itemized), *PI* protease inhibitor, *NNRTI* non-nucleoside reverse transcriptase inhibitor, *INSTI* integrase strand transfer inhibitor, *SD* standard deviation, *IQR* interquartile range. Race/ethnicity and natal sex were self-reported.

### Baseline PCGrimAge, DunedinPACE, and PCDNAmTL and changes over 24 weeks of semaglutide

At baseline, PCGrimAge, an epigenetic biomarker trained to predict mortality risk, indicated a biological age of 60.0 years (median; range: 34.3–70.6). The median PCGrimAge acceleration reflecting the difference between epigenetic and chronological age was 7.5 years (IQR: 5.1–9.4), suggesting elevated age-related mortality risk within the cohort for 40 of the 41 participants profiled. The DunedinPACE score, which quantifies the rate of aging (with 1.0 representing the normative pace), had a median value of 0.95 (IQR: 0.88–1.00). Twenty-two percent (9 of 41 participants) had a DunedinPACE score calculated at greater than 1.0 at baseline, indicating an accelerated pace of aging for these individuals. PCDNAmTL, a methylation-derived estimate of telomere length, showed a median value of 6.90 units (IQR: 6.80–7.16). Over 24 weeks of semaglutide (1.0 mg/week), we observed overall group changes of median DunedinPACE change of +0.018 (IQR: –0.023 to +0.053), change in PCDNAmTL (median –0.006 kb; IQR: –0.073 to +0.054), and change in PCGrimAge (median +0.54 years; IQR: –0.33 to +1.26). In the absence of a control arm, these overall group changes cannot be attributed to treatment versus short-term natural trajectories.

### Associations of epigenetic changes with hepatic and functional outcomes

**Fig. 1:**
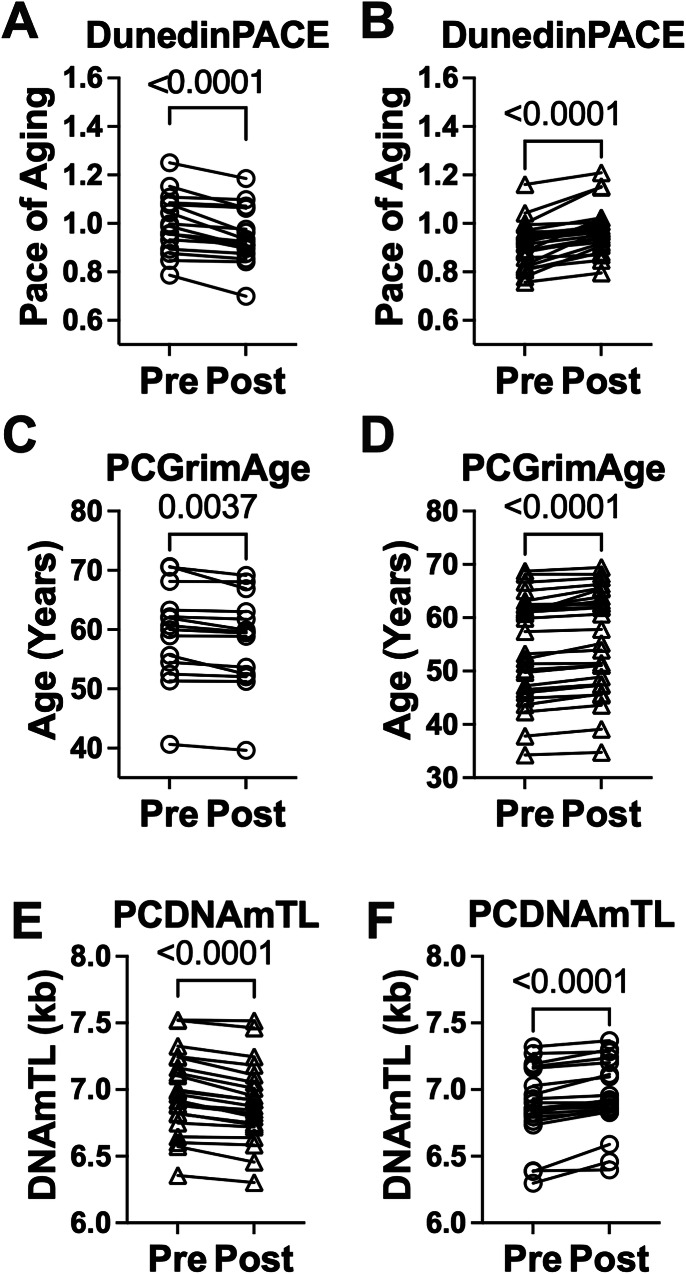
Changes in epigenetic aging biomarkers before and after 24 weeks of semaglutide treatment in people with HIV and MASLD (n = 41), grouped by direction of epigenetic change. **A** DunedinPACE, a measure of the pace of aging (1.0 = normative rate), decreased following treatment with 17 participants (41.5%) demonstrating a reduction indicating a slower pace of aging Paired *T* Test. *P* < 0.001. **B** 24 participants demonstrating an increase in DunedinPACE. Paired *T* Test. *P* < 0.001. **C** PCGrimAge, an epigenetic estimate of biological age and mortality risk, also declined with 14 participants (34.1%) showing reductions in predicted age. Paired *T* Test. *P* = 0.0037. **D** 27 participants (65.8%) showing an increase in PCGrimAge. Paired T Test. *P* < 0.001. **E** 21 (51.2%) participants showing decrease in PCDNAmTL. Paired *T* Test. *P* < 0.001. **F** PCDNAmTL, a methylation-derived estimate of telomere length (in kilobases) increased in 20 participants (48.8%) showing predicted telomere elongation. Paired *T* Test. *P* < 0.001. Circles indicate participants with beneficial changes (slower pace of aging, reduced epigenetic age, or longer DNAm telomere length), while triangles indicate participants with changes in the opposite direction over the 24-week period. Data are shown as paired values with lines connecting participants. These groupings were used to examine whether directional shifts in epigenetic aging over the 24-week period were associated with differential clinical responses to semaglutide, including outcomes related to anthropometry, metabolic biomarkers, and physical function.

A total of 17 participants (9 male, 8 female; 41.5%) experienced a decrease in DunedinPACE with semaglutide, indicating a slower pace of aging from baseline. 14 participants (8 male, 6 female; 34.1%) demonstrated a decrease in PCGrimAge, indicating a reduction in epigenetic mortality risk, and 20 participants (11 male, 9 female; 48.8%) showed an increase in predicted DNA methylation-based telomere length (PCDNAmTL) (Fig. 1A–F).

**Fig. 2:**
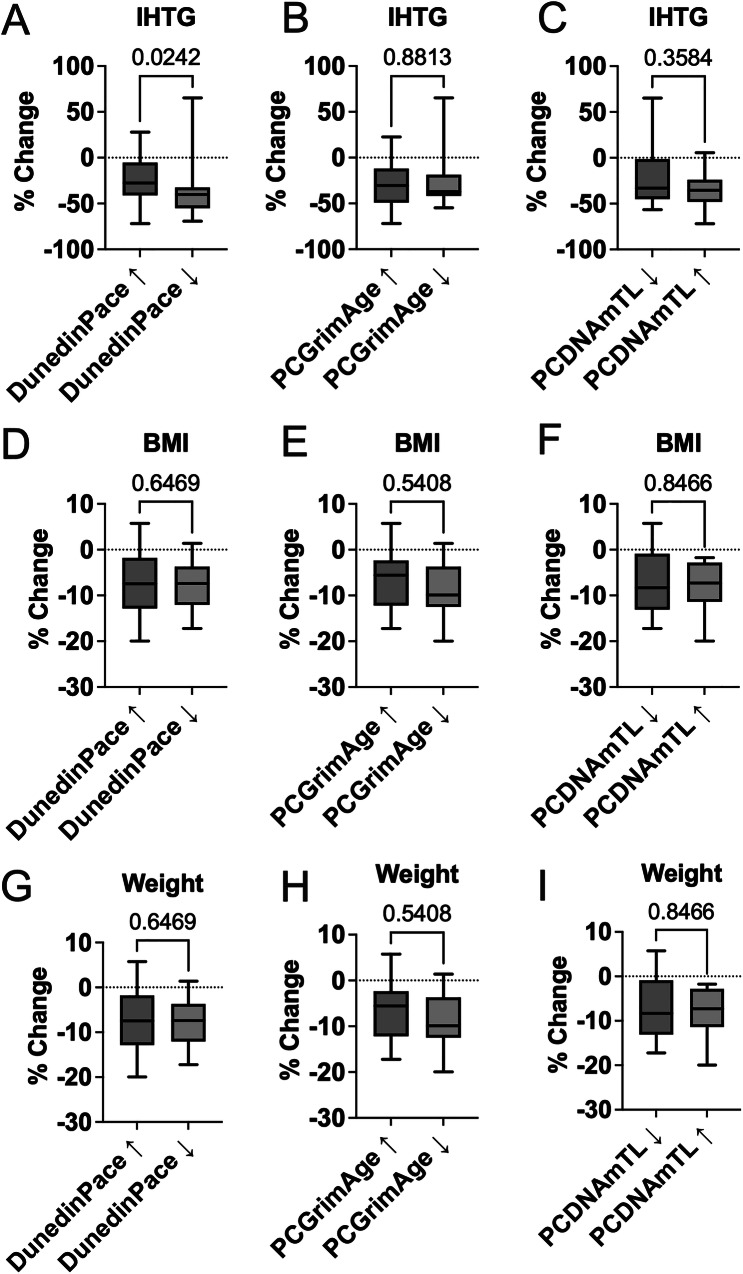
Differences in 24-week changes in IHTG and anthropometric percent change following low-dose (1 mg) weekly semaglutide by epigenetic age change group. Boxplots display the percent change in intrahepatic triglycerides (IHTG) for (**A**) DunedinPace, (**B**) PCGrimAge, and **C** PCDNAmTL stratified by 24 week group direction of change. Boxplots display the percent change in body mass index (BMI) for (**D**) DunedinPace, **E** PCGrimAge, and (**F**) PCDNAmTL stratified by 24 week group changes. Boxplots display the percent change in body weight for (**G**) DunedinPace, **H** PCGrimAge, and **I** PCDNAmTL stratified by 24 week group changes. Each panel shows median, interquartile range, and range of percent changes. Statistical comparisons were conducted using the Mann–Whitney nonparametric test, with unadjusted *p* values annotated above each panel.

We explored whether changes in epigenetic aging over the 24 weeks were associated with differential responses to semaglutide treatment across key clinical domains, including anthropometry, metabolic biomarkers, and physical function. Participants were grouped based on direction of change (Increased vs. Decreased) for DunedinPACE, PCGrimAge, and PCDNAmTL. Participants with a decrease in DunedinPACE showed a greater percent reduction in IHTG (*p* = 0.024) compared to those with increased DunedinPACE (Fig. 2). No nominal differences were observed for BMI (*p* = 0.63) or weight (*p* = 0.63) (Fig. 2). When stratified by PCGrimAge or PCDNAmTL change groups, no nominal group differences were observed for any anthropometric outcome (all *p* > 0.35), including IHTG (*p* = 0.88 for PCGrimAge, *p* = 0.36 for PCDNAmTL), suggesting the IHTG association may be marker-specific to DunedinPACE in this pilot analysis (Fig. 2).

**Fig. 3:**
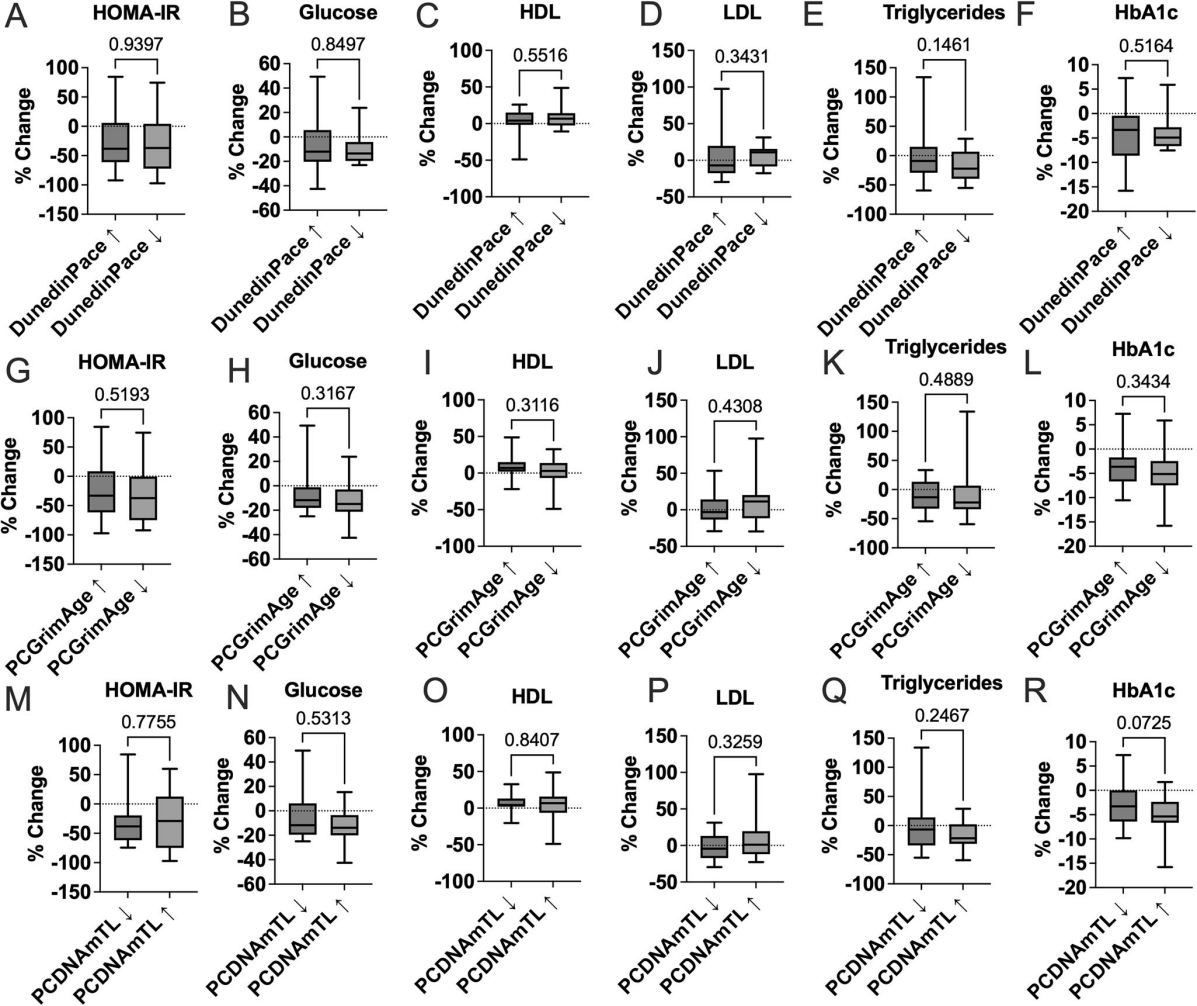
Group differences in lipids and glucose homeostasis percent changes following semaglutide by epigenetic age change. Boxplots display the percent change in (**A**) Homeostatic Model Assessment of Insulin Resistance (HOMA-IR), **B** glucose, **C** high-density lipoprotein (HDL) cholesterol, **D** low-density lipoprotein (LDL) cholesterol, **E** triglycerides, **F** hemoglobin A1c (HbA1c) stratified by 24 week group direction of change for DunedinPACE. Boxplots display the percent change in (**G**) Homeostatic Model Assessment of Insulin Resistance (HOMA-IR), **H** glucose, **I** high-density lipoprotein (HDL) cholesterol, **J** low-density lipoprotein (LDL) cholesterol, **K** triglycerides, **L** hemoglobin A1c (HbA1c) stratified by 24 week group direction of change for PCGrimAge. Boxplots display the percent change in (**M**) Homeostatic Model Assessment of Insulin Resistance (HOMA-IR), **N** glucose, **O** high-density lipoprotein (HDL) cholesterol, **P** low-density lipoprotein (LDL) cholesterol, **Q** triglycerides, **R** hemoglobin A1c (HbA1c) stratified by 24 week group direction of change for PCDNAmTL. Each panel shows median, interquartile range, and range of percent changes. Statistical comparisons were conducted using the Mann–Whitney nonparametric test, with unadjusted *p* values annotated above each panel.

We observed no nominal differences in metabolic biomarkers by change group for DunedinPACE, PCGrimAge, or PCDNAmTL. This included HOMA-IR (*p* = 0.94 for DunedinPACE, *p* = 0.78 for PCDNAmTL), fasting glucose, triglycerides, high-density lipoprotein (HDL), and low-density lipoprotein LDL (all *p* > 0.14), suggesting that semaglutide-induced improvements in these markers occurred broadly and were not contingent on three epigenetic age dynamics assessed (Fig. 3). However, a trend toward greater reduction in HbA1c was observed among participants with increased PCDNAmTL (*p* = 0.072), suggesting a possible relationship between telomere attrition and glycemic improvement (Fig. 3).

**Fig. 4:**
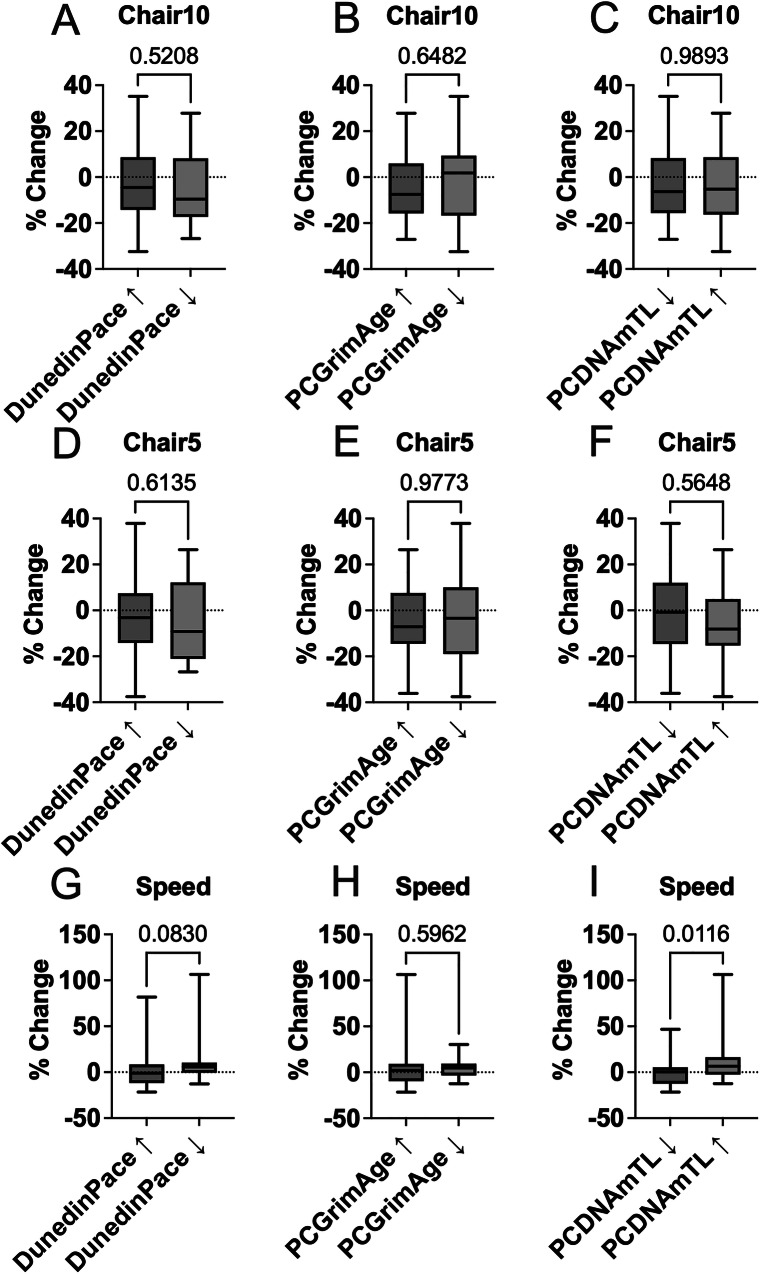
Group differences in physical function percent changes following semaglutide by epigenetic age change. Boxplots display the percent change time to complete 10 chair stands (Chair10) for **A** DunedinPace, **B** PCGrimAge, and **C** PCDNAmTL stratified by 24-week group direction of change. Boxplots display the percent change time to complete 5 chair stands (Chair5) for **D** DunedinPace, **E** PCGrimAge, and **F** PCDNAmTL stratified by 24-week group direction of change. Boxplots display the percent change walking speed for **G** DunedinPace, **H** PCGrimAge, and I PCDNAmTL stratified by 24-week group direction of change. Statistical comparisons were conducted using the Mann-Whitney nonparametric test, with p values annotated above each panel.

For physical function, no nominal differences were found in 5-time or 10-time chair rise time by DunedinPACE, PCGrimAge, or PCDNAmTL group (all *p* > 0.50) (Fig. 4). An improvement in gait speed was observed among participants with increased PCDNAmTL (*p* = 0.012), suggesting that preservation or elongation of telomere length may be linked to better maintenance of physical function following semaglutide treatment. We also observed a trend toward improved gait speed among those with decreased DunedinPACE (*p* = 0.083) (Fig. 4).

## Discussion

In this pilot post hoc epigenetic analysis of the SLIM LIVER trial, we evaluated whether changes in epigenetic aging biomarkers, specifically DunedinPACE, PCGrimAge, and PCDNAmTL, were associated with anthropometric, metabolic, and physical function changes over 24 weeks of semaglutide treatment in PWH and MASLD. Our findings suggest that a less accelerated pace of aging, as captured by DunedinPACE, may be selectively associated with greater liver fat reduction following semaglutide treatment. While PCGrimAge change was not linked to anthropometric or metabolic outcomes, an increase in telomere length (PCDNAmTL) was associated with improved gait speed, indicating that epigenetic telomere preservation may parallel enhancements in physical function. Conversely, participants with increased PCDNAmTL trended toward greater reductions in HbA1c, hinting at a possible link between semaglutide glycemic improvement and telomere biology. These findings suggest a potential relationship between semaglutide-related changes in specific epigenetic aging biomarkers and improvements in liver fat and physical function. These results merit further investigation of this association in larger cohorts and independent studies to validate the utility of epigenetic age biomarkers as indicators of therapeutic response in GLP-1RA therapy.

The observed reduction in liver fat among select individuals with slowing DunedinPACE may reflect improved metabolic flexibility and hepatic lipid mobilization in response to semaglutide. DunedinPACE, is a third-generation DNA methylation-based biomarker that quantifies the pace of biological aging by integrating longitudinal physiological, cellular, and molecular data across multiple organ systems^21^. Unlike first generation epigenetic clocks, it captures short-term changes in systemic function and has been shown to respond to behavioral and pharmacologic interventions^22,23^. Our findings are consistent with prior work demonstrating associations between accelerated DunedinPACE and liver fibrosis severity, insulin resistance, and cardiometabolic risk^17^. The lack of similar associations with body weight or BMI following semaglutide treatment in this study suggests that DunedinPACE may be more sensitive to underlying shifts in tissue-specific metabolic health such as intrahepatic lipid dynamics than to gross anthropometric changes alone. In a 2-year caloric restriction clinical trial there was a modest slowing of the pace of aging by DunedinPACE, with minimal change in other clocks^24^. These findings support further exploration of DunedinPACE as a potential biomarker for semaglutide-related improvements in metabolic aging beyond weight loss alone.

Interestingly, we did not observe group differences in glycemic or lipid parameters when stratified by epigenetic age change following semaglutide. Improvements in HOMA-IR, fasting glucose, and triglycerides were seen broadly across the cohort following semaglutide^12^, regardless of whether DunedinPACE or PCGrimAge declined over the study period. These findings suggest that semaglutide’s core metabolic benefits may operate, at least in part, through mechanisms independent of its impact on the three DNA methylation-based epigenetic measures assessed. Alternatively, the lack of association may reflect limited power to detect domain-specific effects given the modest sample size. Importantly, while semaglutide has demonstrated robust metabolic efficacy, its potential as a multi-system gerotherapeutic is only beginning to be explored^25^. Emerging data suggest that GLP-1 RAs may influence aging biology in other organ systems such as the brain, cardiovascular system, and kidneys via effects on inflammation, oxidative stress, and cellular senescence^7,26^. The SLIM LIVER study provides supporting preliminary evidence that semaglutide may also modulate biological aging trajectories in the liver and musculoskeletal system, as reflected by links to liver fat reduction and gait speed improvement. Future work incorporating broader multi-organ epigenetic and transcriptomic profiling may clarify how GLP-1 RAs influence systemic aging biology beyond metabolic control alone.

A relevant and well-designed prior randomized, double-blind placebo-controlled study of once-weekly semaglutide (1.0 mg) versus placebo for 32 weeks specifically in PWH with HIV-associated lipohypertrophy (without requiring MASLD) reported a substantial reduction in visceral adipose tissue and suggestive findings of reduced liver fat content^7^. Although that study did not explicitly hypothesize to test semaglutide-associated reductions in central adiposity would improve MASLD, its findings support a biologically consistent pathway: reductions in visceral adipose tissue are strongly linked to improvements in hepatic steatosis, systemic inflammation, and metabolic dysfunction in PWH.

We also observed a modest trend toward improved walking speed among participants who exhibited a reduction in DunedinPACE. While this association was exploratory, the directionality is biologically plausible and consistent with prior studies linking accelerated epigenetic aging to frailty, slower gait speed, and functional decline in older adults^27,28^. The absence of group differences in chair rise times may be due to skeletal muscle effects, task variability, or limited power. Taken together, these findings suggest that semaglutide-associated slowing of biological aging may contribute to preserved or enhanced mobility. Future studies in broader populations, more granular functional assessments, and extended follow-up durations are needed to fully characterize the relationship between changes in epigenetic aging markers and physical performance trajectories.

In addition to DunedinPACE and PCGrimAge, we evaluated changes in methylation-derived telomere length (PCDNAmTL). While changes in PCDNAmTL were not associated with differences in hepatic or anthropometric outcomes, we observed a trend toward greater HbA1c reduction among participants with telomere increases, potentially linking glycemic improvement to telomere biology^29^. In addition, participants with increased PCDNAmTL indicating preserved or elongated telomeres demonstrated an improvement in walking speed, suggesting a possible protective effect of telomere maintenance on physical function. In the SLIM LIVER study we found the prevalence of slow gait speed ( < 1 m/sec) decreased from 63% to 46% (P =.029)^11^. These findings align with prior evidence linking longer telomeres to better mobility, mitochondrial integrity, and muscle performance in aging populations^30^. Although exploratory, the directionality of these associations suggests that different dimensions of epigenetic aging may differentially track metabolic versus functional responsiveness to treatment. Larger studies are needed to validate these relationships and clarify whether PCDNAmTL may serve as a prognostic marker for physical resilience in the context of semaglutide.

An important consideration in interpreting these results is the use of a relatively low semaglutide dose (1.0 mg weekly) and a 24-week treatment duration, which may have attenuated the magnitude of clinical effects compared to trials using higher doses over longer periods. For example, phase 3 trials such as the STEP and ESSENCE programs have demonstrated greater reductions in liver fat, weight, and glycemic indices with weekly 2.4 mg dosing over 48 to 72 weeks^1,3,6^. However, for anti-aging applications, prolonged tolerability and safety are critical, and lower, sustained dosing regimens may be more appropriate for long-term use. As such, while higher doses could potentially amplify clinical and epigenetic responses, the current design may reflect a more pragmatic framework for gerotherapeutic implementation. Future studies should explore dose–response effects, compare acute versus chronic trajectories of epigenetic change, and evaluate the durability of biological aging modifications with extended follow-up in target populations, including PWH.

An additional potential confounder relevant to interpretation of these findings is background ART. In the current study, 68% of participants were receiving a tenofovir alafenamide (TAF)–containing regimen, whereas only 7% were on tenofovir disoproxil fumarate (TDF). Prior studies have demonstrated divergent hepatic metabolic effects of these agents, with TAF associated with increased risk of hepatic steatosis and TDF associated with a lower or potentially protective risk of steatosis in PWH^31^. These differences raise the hypothesis that ART composition may influence baseline liver fat burden, epigenetic aging profiles, and responsiveness to GLP-1 receptor agonist therapy. Moreover, antiretroviral drugs may interact with metabolic and inflammatory pathways that underlie both MASLD pathogenesis and DNA methylation–based aging biomarkers^32^. Such pathways overlap with known mechanisms of GLP-1 receptor agonists, including effects on hepatic lipid metabolism, insulin sensitivity, and systemic inflammation^33^. Although the present study was not powered to evaluate differential responses by ART regimen, future studies should explicitly examine potential interactions between TAF and TDF, epigenetic aging trajectories, and treatment response to GLP-1 RAs, particularly in PWH with metabolic complications.

This was an exploratory, post hoc analysis of a single-arm, 24-week trial with a modest sample size, no placebo control, and semaglutide dosed at 1.0 mg/week; together, these factors limit causal inference, reduce power, and may underestimate dose–response effects. Our stratification by biomarker change (decrease vs. increase) introduces potential regression-to-the-mean and dichotomization bias, and multiple comparisons without formal multiplicity control raise the risk of type I error. Epigenetic measures were derived from peripheral blood rather than target tissues (e.g., liver, skeletal muscle), may be influenced by shifts in leukocyte composition, and reflect clock models developed largely outside HIV cohorts; PCDNAmTL is a proxy of telomere length and not a direct measurement. Residual confounding from unmeasured behaviors or medications (e.g., diet, activity, ART changes) cannot be excluded, and the 24-week window precludes conclusions about durability. Finally, generalizability may be limited to PWH with MASLD meeting ACTG A5371 eligibility. Sensitivity analyses (e.g., modeling biomarkers continuously with mixed effects, adjusting for weight change and baseline values, controlling for estimated cell composition, and applying false-discovery rate procedures) and validation in larger, randomized, longer-duration cohorts with varying doses will be important next steps. Because no multiplicity correction was applied, some nominal associations may represent false positives, and results should be interpreted as hypothesis-generating pending confirmation in larger studies.

Taken together, our findings provide preliminary evidence that semaglutide may influence biological aging trajectories in a subset of individuals, particularly through deceleration of the pace of aging as measured by DunedinPACE. This slowing was selectively associated with greater reductions in liver fat, suggesting that epigenetic aging dynamics may reflect or contribute to organ-specific treatment responsiveness. Given the disproportionate burden of metabolic dysfunction in PWH and the expanding therapeutic role of GLP-1RA in liver disease, these results highlight the importance of integrating biological aging metrics into future interventional studies. Epigenetic clocks such as DunedinPACE and DNAmTL may serve as minimally invasive biomarkers to stratify risk, monitor longitudinal response, and guide personalized approaches aimed at improving both metabolic and functional health outcomes.

## Methods

### Trial population

The SLIM LIVER study ([ACTG] protocol A5371; No. NCT04216589, registered 02^nd^ Jan 2020) was a Phase 2b, single-arm, open-label, 24-week, pilot study designed to evaluate the effect of semaglutide on IHTG and metabolic health among PWH and MASLD^11^. Participants were enrolled from nine ACTG-affiliated clinical research sites between February 2021 and September 2022. Eligible participants were aged ≥18 years, living with HIV on stable antiretroviral therapy ART with suppressed HIV-1 RNA ( < 50 copies/mL), had ≥5% IHTG as quantified by magnetic resonance imaging proton-density fat fraction (MRI-PDFF), and demonstrated central adiposity (minimum waist circumference of ≥95 cm for individuals assigned male sex at birth or ≥94 cm for individuals assigned female sex at birth) and either insulin resistance (HOMA-IR > 3.0) or pre-diabetes (fasting glucose 100–125 mg/dL or hemoglobin A1c (HbA1c) 5.7–6.4%). Sixty-eight percent of the current study participants were on a tenofovir alafenamide (TAF)-containing regimen, while 7% were taking a tenofovir disoproxil fumarate (TDF)-containing regimen. Exclusion criteria included previous GLP-1 RA use within 24 weeks, diabetes mellitus, significant alcohol use of ≥ 3 months within 90 days prior to screening (consuming ≥5 alcoholic drinks for men or ≥4 for women during a single occasion or ≥3 drinks on 4 mor more days of the week on average for men or ≥2 drinks on 4 or more days of the week on average for women), other causes of liver disease, known active hepatitis C virus infection defined as a detectable HCV RNA within 24 weeks prior to study entry, and active/chronic hepatitis B defined as a positive hepatitis B surface antigen (HBsAg) at screening. Physical function was measured at baseline and week 24 by assessing the time to rise from a chair 5 and 10 times and 4-meter gait speed, where gait speed was calculated as the average of 2 measurements at usual pace. Slow gait speed was defined as walking <1 m/sec. Each site obtained institutional review board approval, and all participants provided written informed consent. All methods were performed in accordance with the Declaration of Helsinki.

### SLIM LIVER epigenetic sub-study participant selection

We evaluated the longitudinal changes in epigenetic age estimates at two time points, at baseline and after 24 weeks of low-dose semaglutide, for 41 participants enrolled with available peripheral blood mononuclear cells (PBMCs). Selection was driven by availability of paired PBMCs, not clinical outcomes. Baseline characteristics of included participants were highly similar to the per-protocol cohort^33^. Age was stratified as <50 vs ≥50 years to reflect a geroscience/clinical threshold and to balance subgroup sizes. HIV-1 RNA was categorized with “<20 copies/mL” indicating values below the assay lower limit of quantification (LLOQ). CD4 count was grouped as <500 vs ≥500 cells/mm³ based on an established immune-risk threshold. The CD4/CD8 ratio was grouped as <1.0 vs ≥1.0 to capture immune dysregulation. This was a pilot analysis (*n* = 41) without an a priori power calculation.

### DNA methylation profiling and epigenetic age

DNA was isolated from PBMCs using a Zymo Research Quick-DNA microprep kit. 500 ng of DNA was treated with bisulfite using the EZ DNA Methylation kit from Zymo Research, following the manufacturer’s instructions. The bisulfite-treated DNA samples were randomly assigned to a well on the Infinium HumanMethylationEPIC BeadChip, which was then amplified, hybridized, stained, washed, and imaged with the Illumina iScan SQ instrument to obtain raw image intensities. To pre-process the DNA methylation data, we used the *minfi* pipeline^34^, and low quality samples were identified using the *qcfilter()* function from the ENmix package^35^, using default parameters. A total of 82 samples (41 baseline and 41 follow up), representing 100% of the original samples, passed the quality assurance and quality control (p < 0.05) and were deemed to be high quality samples. Our focus was on the second-generation principal component-derived epigenetic clock, PCGrimAge^36,37^, a measure of biological aging that incorporates DNA methylation-based estimates of biomarkers associated with age-related mortality risk, the third-generation clock, DunedinPACE^37^, a measure of the pace of aging crucial for understanding the impact of interventions on epigenetic aging, and Lu’s telomere length predictor based on 140 CpGs^38^. Epigenetic clocks were calculated according to published methods^36^ from processed DNA methylation data. To enhance the reliability of GrimAge and DNAmTL estimates, we utilized its principal-component versions using the custom R script available via GitHub (https://github.com/MorganLevineLab/PC-Clocks)^37^. The pace of aging clock, DunedinPACE, was calculated using the *PACEProjector* function from the DunedinPACE package available via GitHub (https://github.com/danbelsky/DunedinPACE). For DunedinPACE, a − 0.02 to −0.03 shift ( ~ 2–3% slowing) was considered minimally important, with −0.05 ( ~ 5% slowing) a clear target; for PCGrimAge age-acceleration, −0.5 years was considered minimal and ≥−1.0 year a target; for PCDNAmTL (PC score), +0.25 SD was considered minimal and ≥+0.50 SD a target.

### Statistical analysis

This exploratory post hoc analysis assessed whether changes in epigenetic aging markers over 24 weeks of semaglutide treatment were associated with differential responses across hepatic, metabolic, and physical function domains. Given the pilot, post hoc nature and modest sample size (*n* = 41 paired), no adjustment for multiple comparisons was applied. Descriptive statistics were reported as medians with interquartile ranges (IQRs) for continuous variables and frequencies with percentages for categorical variables. Participants were stratified into “decreased” vs. “increased” epigenetic age change groups based on directionality of change in three biomarkers: DunedinPACE, PCGrimAge, and PCDNAmTL. Directionality (increase vs. decrease) was assessed separately for DunedinPACE, PCGrimAge, and PCDNAmTL. Group comparisons for percent changes in clinical measures (e.g., IHTG, HOMA-IR, HbA1c, BMI, physical function) were conducted using the Kruskal-Wallis test. For epigenetic biomarkers, changes from baseline to week 24 were analyzed using Wilcoxon signed-rank tests for within-group comparisons.

## Data Availability

Due to ethical restrictions, study data are available upon request from sdac.data@sdac.harvard.edu with the written agreement of the Advancing Clinical Therapeutics Globally. The epigenetic data from this study was submitted to the NCBI Gene Expression Omnibus (GEO) http://www.ncbi.nlm.nih.gov/geo/.
